# Bilateral Chylothorax as a Unique Presentation of Pancreaticobiliary or Upper Gastrointestinal Cancer

**DOI:** 10.1155/2019/9387021

**Published:** 2019-07-02

**Authors:** Nooraldin Merza, John Lung, Mazin Saadaldin, Tarek Naguib

**Affiliations:** ^1^Department of Internal Medicine, Texas Tech University Health Sciences Center, Amarillo, TX, USA; ^2^School of Medicine, Texas Tech University Health Sciences Center, Amarillo, TX, USA

## Abstract

Chylothorax presents as exudate with lymphocytic predominance and high triglyceride-low LDH levels, usually due to a traumatic disruption of the thoracic duct, possibly iatrogenic. Other causes include malignancy, sarcoidosis, goiter, AIDS, or tuberculosis. Here we present a case of a 66-year-old male who came in with cough and shortness of breath for few weeks. A week earlier, at an ED visit, he was diagnosed with pneumonia based on CT angiogram of the chest without contrast that showed bilateral pleural effusion and bilateral pulmonary infiltrates. The CT-guided placement of bilateral chest tube drained 1160 cc of creamy yellow fluid on the right and 1200 cc of creamy yellow fluid on the left. CT chest/abdomen/pelvis showed bilateral ground-glass opacities within the lungs and possible bony metastasis. A whole-body bone scan showed multiple bony metastatic lesions throughout the skeleton. IR guided bone biopsy suggested upper GI or pancreaticobiliary cancer. Venous ultrasound with Doppler of left upper extremity showed findings suggestive of a nonocclusive DVT of proximal/mid left subclavian vein which is difficult to compress. Eventually, malignancy-related DVT of the left subclavian/brachiocephalic vein was identified as the possible etiology for the bilateral chylothorax.

## 1. Introduction

Pleural effusion is suspected by dullness to percussion, confirmed by imaging, and worked up by thoracentesis [1]. Chylothorax, the finding of chyle in the pleural space, is diagnosed with pleural fluid triglycerides level greater than 110 mg/dL, whereas less than 50 mg/dL excludes it [2]. Chylothorax presents as exudate with lymphocytic predominance with low LDH levels. However, other pleural fluid presentations with exudative or transudative nature and variable triglyceride, cholesterol, and LDH levels were reported in chylothorax [3]. Chylothorax develops most commonly due to a traumatic disruption of the thoracic duct, possibly iatrogenic. Other causes include malignancy, sarcoidosis, goiter, AIDS, and tuberculosis [4–6]. A retrospective review performed in a tertiary referral clinic suggested that surgery or trauma was the most common cause, with lymphoma or other malignancies accounting for only 16.7% of the cases [7]. Case reports in the literature have linked malignant causes of chylothorax to prostate carcinoma [8], gastric adenocarcinoma [9], mesothelioma [10], lymphomas [11–14], small cell lung cancer [15], and chronic lymphocytic leukemia [16]. The overall prognosis for solid tumor-associated chylothorax is poor, while prognosis is good for lymphoma-associated chylothorax if remission can be achieved [17]. We report a case of a patient presenting with bilateral chylothorax due to underlying pancreaticobiliary or upper gastrointestinal cancer.

## 2. Case Presentation

**Figure 1:**
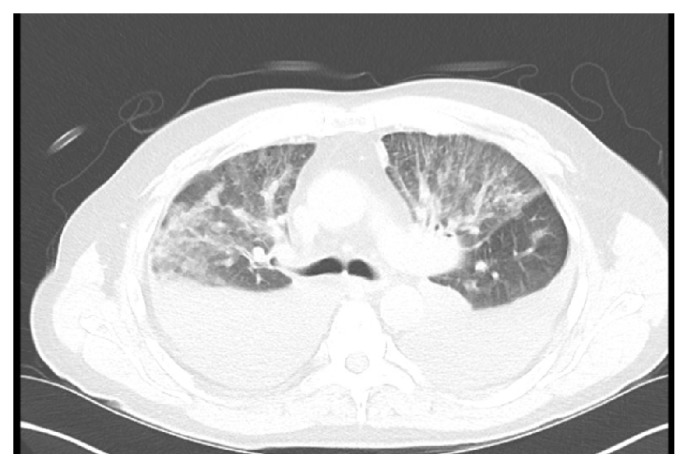
CT of the chest without contrast shows prominent parenchymal ground-glass changes. Large bilateral pleural effusions and moderate pericardial effusion.

A 66-year-old male presented with few weeks of cough and shortness of breath. He had to lay on his side or prop himself up to breathe more comfortably at night. A week earlier, at an ED visit, he was diagnosed with pneumonia based on CT angiogram of the chest without contrast that showed bilateral pleural effusion and bilateral pulmonary infiltrates (Figure 1). Levofloxacin oral therapy was followed by some improvement but he felt worse again. There is no significant past medical or trauma history other than right elbow trauma and right knee endoscopic surgery. He denied tobacco or drug use but endorsed occasional alcohol use. Lungs exam revealed only scant rales in the right lower lobe. He was afebrile, normotensive, and hypoxic with SpO2 of 91% on room air. Lab was only significant for elevated alkaline phosphatase 476, AST 46, and pro-BNP 147. EKG showed normal sinus rhythm. Ceftriaxone and azithromycin were started for pneumonia which failed outpatient therapy.

The CT-guided placement of bilateral chest tube drained 1160 cc of creamy yellow fluid on the right and 1200 cc of creamy yellow fluid on the left. Pleural fluid LDH was 226 units/L, triglycerides were 85 mg/dL, total protein was 4.3 gm/dL, and cholesterol was 67 mg/dL. Total serum protein was 7.8 gm/dL. The fluid was diagnosed as exudative in nature (Light's criteria, pleural fluid protein/serum protein >0.5). The cytopathology evaluation of the pleural fluid was negative.

Antibiotics were stopped due to lack of growth in cultures. A few days after the right-sided chest tube was removed, a chest x-ray showed a recurrent right-sided pleural effusion.

**Figure 2:**
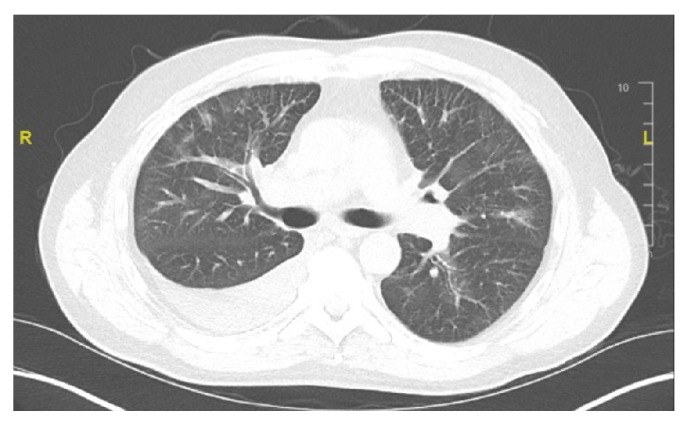
Repeat CT thorax without contrast showing moderate-sized right pleural effusion with right lower lobe atelectasis.

**Figure 3:**
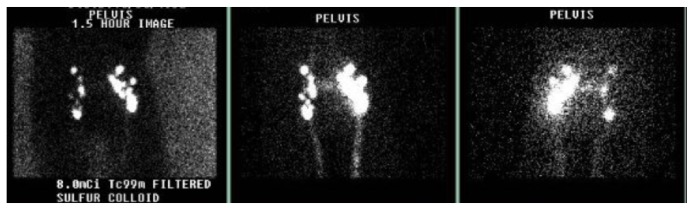
Lymphocytic scintigraphy results of the pelvic area showing no activity beyond the pelvis.

Repeated CT thorax without contrast showed a moderate right-sided pleural effusion with right lower lobe atelectasis (Figure 2). A repeat left pleural fluid analysis showed triglycerides of 1066 mg/dl, LDH of 363 units/L, total protein of 3.6 gm/dL, and cholesterol of 53 mg/dL, highly suggestive of chylothorax. A chest tube was placed again on the right side and octreotide and somatostatin were begun. Lymphocytic scintigraphy (Figure 3) showed no activity transmitted in the thoracic duct beyond the pelvis suggestive of a central obstruction. Numerous enlarged nodes were also seen in the inguinal and iliac areas, concerning for lymphoma.

**Figure 4:**
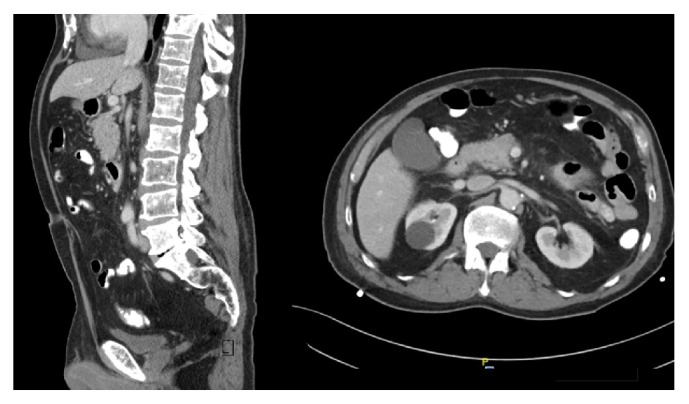
CT chest and abdomen/pelvis with contrast. Diffuse heterogeneous marrow signal of the thoracic spine and lumbar spine (left). Right kidney has several cysts with the largest measuring 2.8 x 2.9 cm (right). Findings were suggestive of diffuse osteopenia versus bony metastasis.

**Figure 5:**
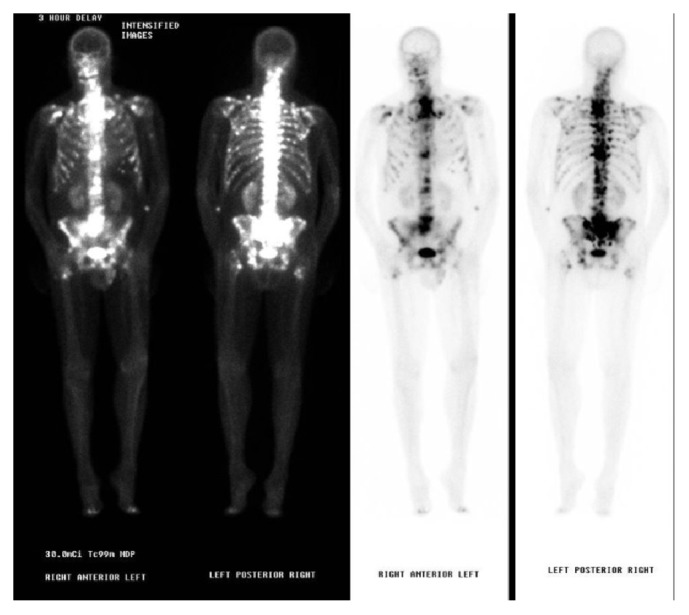
Whole-body bone scan showing multiple bony metastatic lesions throughout the skeleton.

CT Chest/abdomen/pelvis showed bilateral ground-glass opacities within the lungs and possible bony metastasis (Figure 4). A whole-body bone scan showed multiple bony metastatic lesions throughout the skeleton (Figure 5).

IR guided bone biopsy suggested upper GI or pancreaticobiliary cancer (patient's bone marrow biopsy showed poorly differentiated metastatic carcinoma cells but could not completely pinpoint to the site of origin but suggested pancreaticobiliary or upper gastrointestinal cancer). Other work-ups including peripheral blood flow cytometry, SPEP, IFE, and free light chain assay, TSH/free T4, serum quantiferon, ANA, CEA, and serum VEGF all were negative, while CA 19-9 was 402 units/mL.

**Figure 6:**
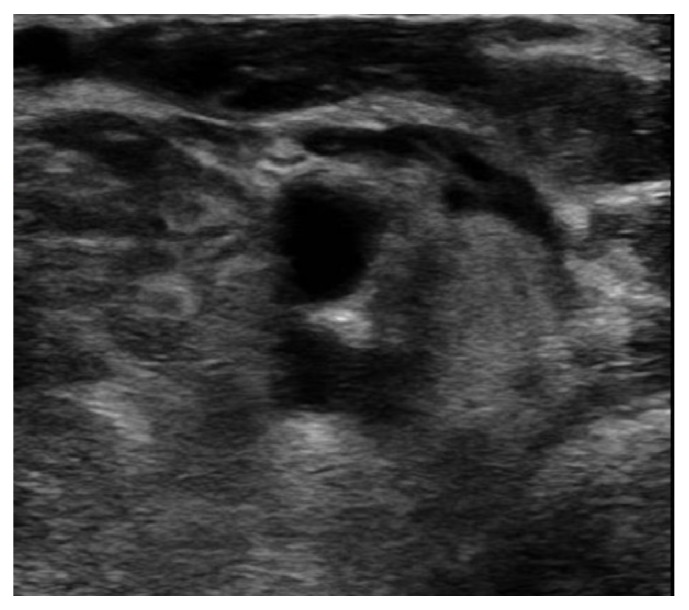
Venous ultrasound with Doppler of left upper extremity showing findings suggestive of a nonocclusive DVT of proximal/mid left subclavian vein which is difficult to compress.

We found that the patient had a deep vein thrombosis of the left subclavian/brachiocephalic vein, which most likely caused the bilateral chylothorax and was confirmed by CTA and upper extremity Doppler ultrasound (Figure 6).

Intravenous heparin was started, followed by Catheter Associated Thrombolysis (EKOS) of left subclavian vein. The heparin drip then switched to apixaban. After the patient's thrombolysis of brachial/subclavian left upper extremity vein was done, his chest tube output started to decrease. Left-sided chest tube was removed four days after the thrombolysis followed by right-sided chest tube seven days after the thrombolysis right before the patient was discharged for outpatient oncology care.

## 3. Discussion

High-volume chylothorax can present with nonspecific symptoms like upper respiratory infection. Rapid onset high volume chylothorax can present with dyspnea, hypovolemia, chest pain, and cough. Chylothorax is typically diagnosed in the context of pleural effusion with triglycerides content of more than 110 mg/dL diagnostic [3].

In our patient, the diagnosis was hampered by an inconclusive first sample from the chest tube. However, the pleural effusion was large enough where the chest tubes drained more than 1L each of creamy yellow fluid. After recurrence, repeated analysis showed triglycerides greater than 1000 mg/dL, suggestive of a bilateral chylothorax. Eventually, malignancy-related DVT of the left subclavian/brachiocephalic vein was identified as the possible etiology for the bilateral chylothorax. A deep vein thrombosis of the left subclavian vein can lead to pressure in return of the thoracic duct, which can cause leakage into the pleural space [18]. The thoracic duct will drain into the left side of the neck 92-95% of the time, with the final termination site varying between the left subclavian vein and internal jugular vein junction, the internal jugular vein, the external jugular vein, and subclavian vein [19].

In patients with chylothorax, once traumatic injuries have been ruled out, malignancy should be explored. If the initial CT fails to determine the cause, lymphatic imaging should be considered. Lymphoscintigraphy has shown good diagnostic utility in visualizing abnormal lymphatic flow in several case reports [20–22].

Management of chylothorax is dependent on the etiology and rate of accumulation of chyle. Initial measures include placement of chest tubes with medication adjuncts such as octreotide and somatostatin (to minimize lymphatic fluid excretion, increase splanchnic arteriolar resistance, and decrease GI flow which decreases lymphatic flow).

Surgical treatment can be done when conservative management fails. The most common surgical treatment is thoracic duct ligation but the mortality and complication rates can be as high as 25% and 38.3%, respectively [23, 24].

Video-Assisted Thoracoscopic Surgery (VATS) has also increased in popularity for treatment. Nonsurgical causes of chylothorax rarely result in surgical intervention [25]. In our case, the patient's chylothorax resolved once interventional radiology performed thrombolysis of the left subclavian vein with balloon angioplasty. The uniqueness of this case comes from the diagnostic challenge and the approach, beside paucity of the reported cases in literature, as we found only 14 cases of chylothorax with underlying gastric carcinoma shown in the summarized table (Table 1) [9, 26–38]. Our case is unique among gastric malignancy-associated chylothorax in that a malignancy-related DVT caused the chylothorax. Other cases of gastric chylothorax were due to metastasis around the thoracic duct or lymphangitis leading to left internal jugular vein narrowing.

A higher suspicion of a malignant cause of the chylothorax can provide prompt diagnosis and shorten the length of stay. Repeat thoracentesis for pleural fluid analysis is encouraged whenever resolution of effusion is slow with conservative management.

## Figures and Tables

**Table 1:** The literature search revealed 14 cases of chylothorax with underlying gastric carcinoma.

Case	Age	Gender	Cancer site	Bilateral	LDH	Triglycerides	Proteins	Cause of thoracic duct blockage
				or unilateral				
Segal et al., 1986	69	Male	Gastric carcinoma	Bilateral	69 U/L	237 mg/dL	4.3 g/dL	Metastasis

Bautz et al., 1991	38	Female	Signet ring cell carcinoma of stomach	Bilateral	266 U/L	238 mg/dL^**1**^	3.9 g/dL	Metastasis

Shibata et al., 1998	58	Female	Signet ring cell carcinoma of stomach	Bilateral	133 U/L	673 mg/dL	3.4 g/dL	Metastasis

Mogulkoc et al., 1999	19	Female	Signet ring cell carcinoma (unknown source)	Bilateral	230 U/L	335 mg/dL	2.8 g/dL	Metastasis

Yamada et al., 2001	58	Female	Poorly differentiated gastric adenocarcinoma	Bilateral	190 U/L	115 mg/dL	7.0 g/dL	Metastasis

Watanabe et al., 2004	66	Female	Signet ring cell carcinoma of stomach	Bilateral	76 U/L	1045 mg/dL	3.6g/dL	Metastasis

Miyazaki et al., 2007	64	Male	Poorly differentiated gastric adenocarcinoma	Bilateral	113 U/L left, 118 U/L right	187 mg/dL left, 432 mg/dL right	4.0 g/dL Left, 4.1 g/dL Right	Metastasis

Majoor et al., 2007	64	Male	Gastric carcinoma	Unilateral	122 U/L	38.2 mmol/L	31.4 g/L	Metastasis

Kayacan et al., 2008	28	Female	Signet ring cell carcinoma of stomach	Bilateral	7.22 *μ*kat/L	1.65 mmol/L	33 g/L	Metastasis

Miwa et al., 2009	77	Female	Signet ring cell carcinoma of stomach	Bilateral	239 U/L	263 mg/dL	3.9 g/dL	Metastasis

Yoshizawa et al., 2013	61	Female	Signet ring cell carcinoma of stomach	Bilateral	605 U/L	958 mg/dL	5.8 g/dL	Metastasis

Devaraj et al., 2014	23	Male	Signet ring cell carcinoma of stomach	Bilateral	172 U/L	274 mg/dL	3.4 g/dL	Narrowing of IJV, lymphangitis

Wu et al., 2016	63	Female	Poorly differentiated gastric adenocarcinoma	Bilateral	136.5 U/L	4.43 mmol/L	Not reported^**2**^	Metastasis

Tsuji et al., 2018	58	Male	Poorly differentiated gastric adenocarcinoma	Bilateral	Not Reported	913 mg/dL	Not reported	Metastasis

^1^Taken on readmission 3 weeks after initial presentation.

^2^Not reported, 25.1 g/L albumin reported.
